# Specialized Pro-Resolving Lipid Mediators: New Therapeutic Approaches for Vascular Remodeling

**DOI:** 10.3390/ijms23073592

**Published:** 2022-03-25

**Authors:** Lucía Serrano Díaz del Campo, Raquel Rodrigues-Díez, Mercedes Salaices, Ana M. Briones, Ana B. García-Redondo

**Affiliations:** 1 Departamento de Farmacología, Instituto de Investigación Hospital La Paz, Universidad Autónoma de Madrid, 28029 Madrid, Spain; lucia.srrn5@gmail.com (L.S.D.d.C.); raquel.rodrigues@uam.es (R.R.-D.); mercedes.salaices@uam.es (M.S.); ana.briones@uam.es (A.M.B.); 2 Centro de Investigación Biomédica en Red de Enfermedades Cardiovasculares (CIBERCV), 28029 Madrid, Spain; 3 Departamento de Fisiología, Instituto de Investigación Hospital La Paz, Universidad Autónoma de Madrid, 28029 Madrid, Spain

**Keywords:** aneurysm, atherosclerosis, inflammation, remodeling, resolution, restenosis

## Abstract

Vascular remodeling is a typical feature of vascular diseases, such as atherosclerosis, aneurysms or restenosis. Excessive inflammation is a key mechanism underlying vascular remodeling via the modulation of vascular fibrosis, phenotype and function. Recent evidence suggests that not only augmented inflammation but unresolved inflammation might also contribute to different aspects of vascular diseases. Resolution of inflammation is mediated by a family of specialized pro-resolving mediators (SPMs) that limit immune cell infiltration and initiate tissue repair mechanisms. SPMs (lipoxins, resolvins, protectins, maresins) are generated from essential polyunsaturated fatty acids. Synthases and receptors for SPMs were initially described in immune cells, but they are also present in endothelial cells (ECs) and vascular smooth muscle cells (VSMCs), where they regulate processes important for vascular physiology, such as EC activation and VSMC phenotype. Evidence from genetic models targeting SPM pathways and pharmacological supplementation with SPMs have demonstrated that these mediators may play a protective role against the development of vascular remodeling in atherosclerosis, aneurysms and restenosis. This review focuses on the latest advances in understanding the role of SPMs in vascular cells and their therapeutic effects in the vascular remodeling associated with different cardiovascular diseases.

## 1. Introduction

Cardiovascular diseases are the leading cause of death worldwide, resulting in around 18 million deaths per year (WHO, 2020).

Endothelial dysfunction is a key early step in the development of several cardiovascular pathologies, including atherosclerosis, coronary artery disease and hypertension among others [1,2,3]. Although the main functional feature of endothelial dysfunction is the impairment of endothelium-dependent vasodilation and vascular hypercontractility, endothelial dysfunction is also characterized by the overexpression of proinflammatory, proliferative and procoagulant molecules that promote vascular remodeling, which, ultimately, affects vessel wall function [4]. Both endothelial dysfunction and vascular remodeling are key events in cardiovascular diseases; therefore, a complete understanding of the underlying mechanisms is fundamental.

Inflammation plays a central role in the pathogenesis of cardiovascular diseases, such as atherosclerosis, aortic aneurysm, balloon angioplasty or intravascular stenting [5,6,7,8,9]. After a vascular insult (mechanical, hemodynamic or metabolic), activated endothelial cells (ECs) promote the recruitment and infiltration of inflammatory circulating cells, including leukocytes, monocytes and T-lymphocytes to the intima. Both inflammatory cells and activated ECs release inflammatory cytokines, chemokines, proliferative growth factors and reactive oxygen species (ROS) that activate vascular smooth muscle cell (VMSC) proliferation and migration. Moreover, this inflammatory milieu promotes the synthesis and accumulation of extracellular matrix (ECM) by activated VMSCs, resident fibroblasts and infiltrated macrophages, which also contributes to vascular remodeling [10]. Termination of inflammation, known as resolution of inflammation, is a critical process in the restoration of tissue homeostasis [11]. Anti-inflammation is not equivalent to resolution. While anti-inflammation limits the actions of proinflammatory mediators, resolution activates specific mechanisms to return to homeostasis. Resolution is mediated by a family of specialized pro-resolving mediators (SPMs) that limit immune cell infiltration and initiate tissue repair mechanisms [12]. Therefore, failure to resolve inflammation will provoke prolonged inflammation and profound changes in the affected tissues. In this regard, increasing evidence indicates that lack of resolution might be a major cause of the pathophysiology of commonly occurring vascular diseases, including atherosclerosis, aortic aneurysm and neointimal hyperplasia [13]. Therefore, addressing specific biochemical and cellular mechanisms involved in the resolution of inflammation at the vascular level would provide a framework for investigating resolution-based pharmacology and disease mechanisms. This review summarizes current knowledge of SPM actions in vascular cells and their role in vascular remodeling.

## 2. Vascular Remodeling

Vascular remodeling refers to changes in vascular structure to maintain adequate blood flow to the tissues [14]. Usually, vascular remodeling is a response to hemodynamic changes, such as pregnancy or ageing. However, vascular remodeling can become maladaptative, as in atherosclerosis, restenosis, aneurysm, hypertension or obesity. Underlying mechanisms involved in vascular remodeling include alterations in phenotype, migratory capacity or death of VSMCs, as well as changes in the synthesis, reorganisation or degradation of ECM proteins [6,15].

**Figure 1:**
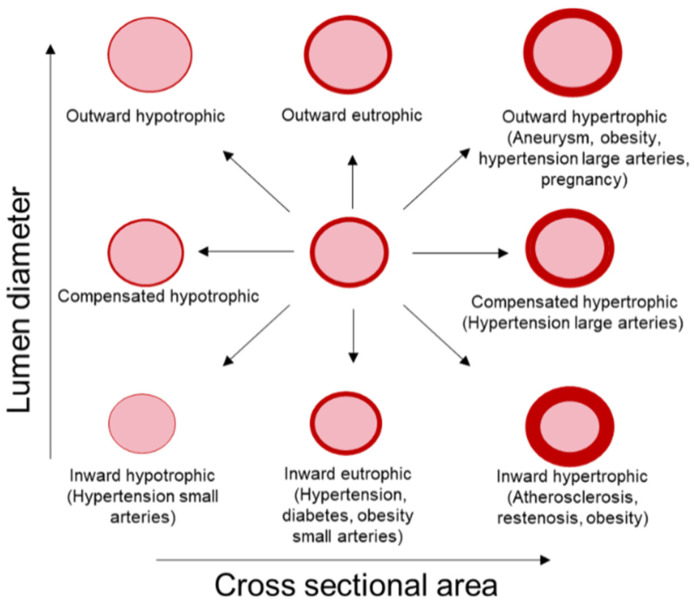
Types of vascular remodeling. Vascular remodeling includes changes in lumen diameter and/or vessel cross-sectional area (CSA). Remodeling can be inward (smaller lumen diameter), outward (greater lumen diameter) or compensated (unaltered lumen diameter). According to changes in vessel CSA, remodeling can be classified as eutrophic (unaltered CSA), hypotrophic (decreased CSA) or hypertrophic (augmented CSA). Modified from [16].

Vascular remodeling may involve an increase (outward), a decrease (inward) or no change (compensated) in vessel size. It can also be classified as hypertrophic, hypotrophic or eutrophic when associated with an increase, a decrease or no change in vessel wall material, respectively [16,17] (Figure 1). Hypertrophic remodeling is observed in large arteries with aging and hypertension [18,19], atherosclerosis and restenosis and predominates in secondary hypertension [20]. This type of remodeling is typically characterized by an increased cross-sectional area and/or media thickness due to increased proliferation and/or migration of VSMCs [16]. Hypotrophic remodeling has been found in the small arteries of some animal models of hypertension [21,22] and is due to apoptosis or a rearrangement of the material of the vascular wall [23]. Finally, eutrophic remodeling is observed in small arteries of patients with hypertension and in animal models of hypertension, diabetes or obesity [18,19]. A combination of inward vascular wall growth and apoptosis in the peripheral area or prolonged vasoconstriction are the main mechanisms described for this type of remodeling [18]. Likewise, outward remodeling occurs in aneurysms, obesity, in large arteries during hypertension and in uterine arteries during pregnancy, while inward remodeling can be observed in obesity, atherosclerosis, restenosis or resistance arteries in hypertension [19,24] (Figure 1).

Underlying mechanisms involved in vascular remodeling include hemodynamic, metabolic and inflammatory factors, though it is not the aim of this study to review this aspect in detail. Although vascular remodeling observed in atherosclerosis, aneurysms and restenosis is clearly different, common mechanisms include as activation of the endothelium, phenotypical changes in VSMCs, alteration in ECM deposition and infiltration of immune cells. In general, in response to a variety of stimuli, including ROS, inflammatory cytokines, LDL accumulation, mechanical forces and catecholamines, ECs express chemokines, selectins and adhesion molecules that facilitate the adhesion, rolling and transmigration of immune cells, such as macrophages and T-lymphocytes [7,9,25]. In this context, ECs can also secrete proinflammatory mediators, such as IL-6, IL1β or TNF-α, and thus contribute to the proinflammatory microenvironment in the vascular wall. Interactions between ECM and vascular cells are crucial for normal vessel function. Inflammation or alteration in ECM content affects the plasticity of VSMCs by changing their quiescent phenotype to a pathological (synthetic, osteogenic, macrophage or senescent) phenotype [26]. Both VSMC phenotype switching and macrophage infiltration induce the aberrant deposition of ECM that perpetuates the cycle. Interestingly, not only vascular cells contribute to vascular inflammation; perivascular adipose tissue (PVAT) can release different factors, such as interleukins, cytokines, ROS or angiotensin II (Ang II), which impact vascular structure. In fact, in atherosclerosis or aneurysms, dysregulated PVAT contributes to the infiltration of immune cells and to the maintenance of vascular inflammation [27,28,29]. Therefore, decreasing vascular inflammation might have beneficial effects in vascular remodeling in different cardiovascular pathologies.

## 3. Specialized Pro-Resolving Lipid Mediators (SPMs)

**Figure 2:**
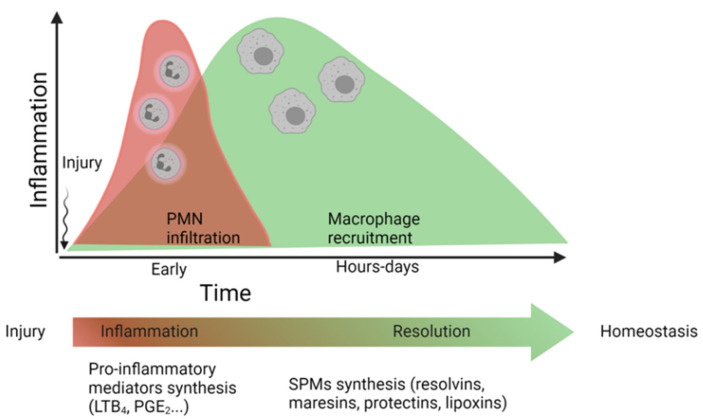
Simplified timeline of the “lipid mediator class switch”. During the first hours after injury, there is an increase in the production of pro-inflammatory mediators (like leukotriene B_4_, (LTB_4_), which act as polymorphonuclear cell (PMN) chemoattractants, as well as factors that increase vascular permeability (i.e., prostaglandin E_2_, PGE_2_). Subsequently, these inflammatory mediators will be replaced by specialized pro-resolving lipid mediators (SPMs) that terminate the inflammatory process and restore tissue homeostasis. Modified from [11].

Transition from inflammation to resolution is marked by a temporal “lipid mediator class switch”. The early phase of an acute inflammatory response is characterized by biosynthesis of pro-inflammatory mediators, such as leukotriene B_4_ (LTB_4_), which is a potent chemoattractant for neutrophils [30,31], or prostaglandin E_2_ (PGE_2_), which increases vascular permeability, and both are key mediators of vascular remodeling in different cardiovascular diseases [32,33,34,35]. These proinflammatory mediators reach a peak at approximately 4 h post injury. Subsequently, they are replaced by SPMs, which limit further polymorphonuclear cell (PMN) infiltration and initiate resolution [12,36,37] (Figure 2). These potent bioactive mediators were first identified by lipidomic analysis in self-resolving inflammatory exudates in mice [38].

SPMs include four structurally unique different families: lipoxins, resolvins, protectins and maresins. The biosynthesis and vascular actions of these families are overviewed herein.

### 3.1. Biosynthesis of SPMs

SPMs are generated from different essential omega-6 and omega-3 polyunsaturated fatty acids (PUFAs): arachidonic acid (AA), eicosapentaenoic acid (EPA), docosahexaenoic acid (DHA) and docosapentaenoic (n-3DPA) acid.

**Figure 3:**
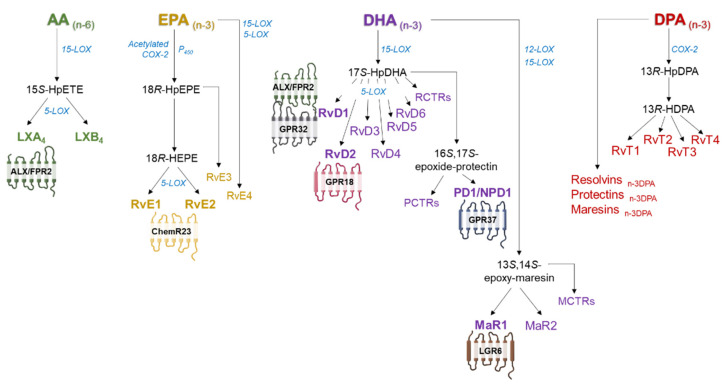
Simplified scheme of the synthesis of specialized pro-resolvin lipid mediators (SPMs) from n-6 and n-3 polyunsaturated fatty acids and the main receptors involved in SPM actions. AA: arachidonic acid, EPA: eicosapentaenoic acid, DHA: docosahexaenoic acid, DPA: docosapentaenoic acid. LX: lipoxin, Rv: resolvin, PD/NPD: protectin/neuroprotectin, MaR: maresin, RvT: 13-series resolvins. GPR: G protein-coupled receptors, LGR6: leucine-rich repeat containing GPR 6, ChemR23: chemerin receptor 23, MCTR: maresin conjugates in tissue regeneration, RCTR: resolvin conjugates in tissue regeneration, PCTR: protectin conjugates in tissue regeneration.

Lipoxins were the first SPM family to be characterized with a dual profile: anti-inflammatory and pro-resolutive. In humans, they are biosynthesized from omega-6 PUFA AA and oxygenated via 15-lipoxygenase (LOX), resulting in 15-hydroxyeicosatetraenoic acid (15*S*-HpETE), which is subsequently rapidly converted via 5-LOX in neutrophils into two different regioisomers: lipoxin A_4_ (LXA_4_) and LXB_4_ [39] (Figure 3). Alternatively, lipoxins can also be biosynthesized in the leukocyte:platelet interaction, where LTA_4_ is converted via 12-LOX into lipoxins [39]. 

The rest of the SPM families are derived from omega-3 PUFA DHA, EPA and DPA. Resolvins are divided into two series depending on the PUFA from which they are biosynthesized. E-series resolvins are biosynthesized from EPA via acetylated cyclooxygenase-2 (COX-2) or cytochrome P450, which synthesizes an 18-hydroperoxide intermediate, 18(R)-HpEPE, subsequently converted into 18R-hydroxyeicosapentaenoic acid (18R-HEPE), which is also a bioactive metabolite. This compound will be finally transformed via 5-LOX to the members resolvin E1 (RvE1) or E2 (RvE2) through leukocyte–endothelial interactions [11]. The 18(R)-HpEPE intermediate is also the precursor to RvE3, which does not require 5-LOX for its conversion (Figure 3) [40]. A new E-series resolvin member, termed RvE4, has been recently discovered. This novel bioactive molecule is biosynthesized in physiological hypoxia by human neutrophils and macrophages via 15- or 5-LOX [40].

DHA is the precursor for a 17-hydroperoxide product, 17S-HpDHA, that is subsequently converted to resolvins D1–D6 in the presence of 5-LOX by human PMNs and macrophages (Figure 3). Specifically, RvD1 and RvD2, the main members of this family, are biosynthesized from a 7S,8S epoxide which is then hydrolyzed to produce RvD1 or D2 [11]. Apart from being the precursor of D-series resolvins, 17S-HpDHA intermediate is also a precursor to 16S,17S-expoxide-protectin, which is transformed to protectin D1 (PD1) by neutrophils, macrophages, eosinophils and T-cells. When produced by neural cells and retinal-pigmented epithelial cells, this mediator is termed neuroprotectin D1 (NPD1/PD1) (Figure 3) [41]. Maresins are also biosynthesized from DHA, but via 12-LOX by macrophages. Subsequently, a 13S,14S-epoxide intermediate (13,14-epoxy maresin) is enzymatically converted to maresin 1 and 2 (MaR1, MaR2) (Figure 3) [41].

Aspirin acetylation of COX-2 leads to 15R-HETE, 18R-HEPE and 17R-HDHA, intermediates of the AA, EPA and DHA pathways respectively, which produce 17R epimers coined as aspirin-triggered (AT) lipoxins, AT resolvins and AT protectins [11]. 

n-3 DPA is less well studied compared to EPA and DHA but is also a substrate and precursor for three families of SPMs: resolvins_n-3 DPA_, protectins_n-3 DPA_ and maresins_n-3 DPA_, with potent pro-resolving effects in human physiology [41,42]. Other studies have shown that during neutrophil–endothelial cell interactions, 13-series resolvins (RvTs) are biosynthesized from n-3 DPA via COX-2 S-nitrosylation [43] (Figure 3). 

Along with the SPMs mentioned above, three families of sulphido-conjugated mediators and their biosynthetic pathways have been identified, named maresin conjugates in tissue regeneration (MCTR), protectin conjugates in tissue regeneration (PCTR) and resolvin conjugates in tissue regeneration (RTCR). These SPMs are specifically relevant in tissue regeneration and repair [44]. DHA is the common precursor of these three new families, 15-LOX being the initiating enzyme in the RCTR and PCTR biosynthetic pathways and 14-LOX the initiating enzyme in MCTR biosynthesis [44]. 

Biosynthesis and transformation into the final lipid mediator can take place within a single cell (e.g., macrophages, PMNs) or during cell–cell interactions, such as leukocyte–endothelium, leukocyte–epithelium or PMN–macrophage interactions. For instance, the intermediate 18-HEPE is generated in ECs and subsequently transformed by leukocytes via 5-LOX to generate RvE1 [38]. MaR1 is produced by circulating neutrophil–platelet aggregates via DHA conversion into 13S, 14S-MaR by the platelet 12-LOX [45]. LXs are generated by PMNs from 15-HETE donated by lung, oral and gastrointestinal epithelial cells [46].

### 3.2. Receptors for SPMs

SPMs produce their biological actions through binding to G protein-coupled transmembrane superfamily receptors (GPCR) (Figure 3). So far, seven receptors for SPMs have been identified: ALX–FPR2 for lipoxin A_4_ and RvD1; DRV1/GPR32 for RvD1; DRV2/GPR18 for RvD2; ERV1/ChemR23 mainly for RvE1 but also for RvE2 [5,47]; GPR37, the potential receptor for NPD1/PD1 [48]; and LGR6, which binds MaR1 [49]. Finally, it has been reported that GPR101 mediates the leukocyte-directed actions of RvD5_n-3DPA_ [50]. In addition, the DRV1/GPR32 receptor is also activated by other D-series resolvin members, such as RvD3 and RvD5, and AT resolvins [51,52]. Moreover, RvE1 and E2 and MaR1 can bind the LTB_4_ receptor 1 (BLT1) with lower affinity, inducing anti-inflammatory responses [5,53,54,55,56].

**Figure 4:**
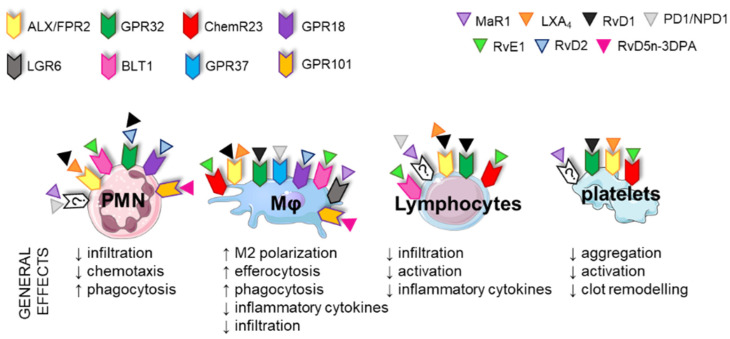
Main receptors for specialized pro-resolving lipid mediators (SPMs) in immune cells and platelets. The key effects of SPMs in immune cells are to decrease inflammation, chemotaxis and infiltration and increase phagocytic activity. SPMs also affect platelet function, decreasing aggregation, activation and clot remodeling. PMNs: polymorphonuclear cells, M_Φ_: macrophage, LX: lipoxin, Rv: resolvin, MaR: maresin.

**Figure 5:**
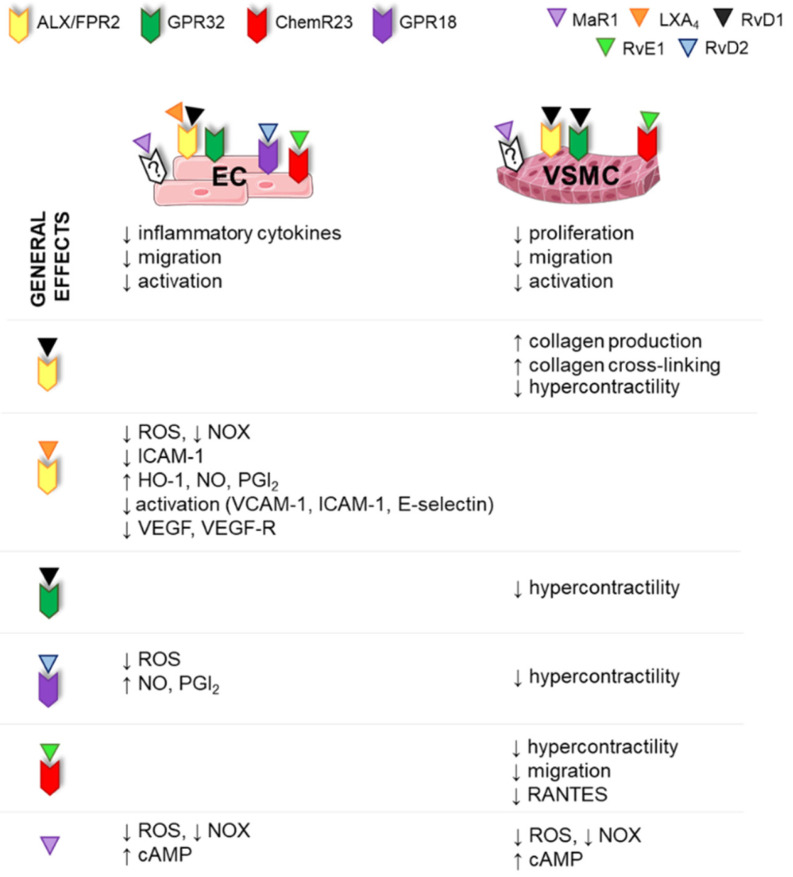
SPM receptors in endothelial cells (ECs) and vascular smooth muscle cells (VSMCs) and key effects of specialized pro-resolving lipid mediators (SPMs) in vascular cells. In general, SPMs decrease activation, migration and inflammatory cytokine release and reactive oxygen species (ROS) production by ECs and VSMCs and increase collagen production by VSMCs. In addition, SPMs act on vascular function decreasing hypercontractility. LX: lipoxin, Rv: resolvin, MaR: maresin, NOX: NADPH oxidase, ICAM-1: intercellular adhesion molecule 1, VCAM-1: vascular cell adhesion molecule, HO-1: hemooxygenase 1, NO: nitric oxide, PGI_2_: prostacyclin, VEGF: vascular endothelial grow factor.

SPM receptors are expressed in different cell types, leading to tissue selectivity (Figure 4 and Figure 5). The GPR32 receptor is expressed in human PMNs, macrophages, T-cells, VSMCs and ECs. ALX–FPR2 is expressed in immune and vascular cells and also in adipocytes and bone marrow cells [54]. GPR18 has been found mainly in immune cells but also in isolated arteries [57] and in EC [58]. ChemR23 is expressed in NK cells, dendritic cells and macrophages [53,59], murine cardiomyocytes [60], human epithelial cells [36] and ECs and VSMCs [61,62,63]. In addition to PMNs and macrophages, BLT1 is also expressed in eosinophils, differentiated T-cells and osteoclasts [64]. GPR37 is expressed in macrophages [48].

### 3.3. Immune Functions of SPMs

SPMs have dual actions, as they possess broad pro-resolving and anti-inflammatory actions in immune cells at the transcriptional and translational levels. In general, these mediators play pivotal roles in different immune responses, including augmented macrophage phagocytosis and efferocytosis, promotion of M2 or “resolutive” macrophage phenotypes, limitation of further leukocyte infiltration and counter-regulation of pro-inflammatory chemical mediators [65,66] (Figure 4). These events will finally lead to resolution of inflammation, reduced organ fibrosis, enhanced wound healing and restored homeostasis. As has already been mentioned, immune cells express several SPM receptors, making the effects of specific SPMs very complex and sometimes overlapping (Figure 4). For example, GPR18 activation reduces infiltration of PMNs, decreases inflammation and increases M2 polarization, efferocytosis and phagocytosis [67]. RvE1, through ChemR23, reduces levels of proinflammatory cytokines and immune cell infiltration and stimulates phagocytosis/efferocytosis in NK cells, dendritic cells and macrophages [53]. GPR37 is expressed in macrophages and its activation by NPD1 enhances macrophage phagocytic activity and resolution of inflammatory pain [48]. Moreover, exogenous SPMs have been found to reduce T-cell recruitment and infiltration into target organs and decrease activation and inflammatory cytokine production [68,69]. Interestingly, human platelets also express the SPM receptors GPR32, ALX–FPR2 and ChemR23, which modulate aggregation, clot remodeling and platelet–PMN interactions. Thus, in general, SPMs have beneficial antithrombotic properties, inhibit inflammatory responses induced by platelet-activating factor and avoid aggregation [70,71,72,73] (Figure 4). These findings suggest that, in principle, any clinical condition that is characterized by immune cell infiltration or altered platelet reactivity might benefit from the effects of SPMs.

### 3.4. Vascular Functions of SPMs

Although the effects of SPMs have been more extensively studied in immune cells, recent evidence demonstrates that vascular cells express SPM receptors, and a range of direct effects of SPMs have been found in the vasculature which ultimately lead to a reduction in their inflammatory state and restore their quiescent state (Figure 5).

#### 3.4.1. SPM Effects in ECs

The endothelium is directly involved in the passage of macromolecules and immune cells and thereby plays a central role in the early inflammatory response. In addition, ECs regulate blood flow, control leukocyte and platelet interaction with the vessel wall and modulate vascular tone by releasing vasodilator and vasoconstrictor factors. Morphological or functional endothelial abnormalities lead to decreased vasodilation and proinflammatory and prothrombic states. Indeed, permanently activated ECs are characterized by expressing cell surface adhesion molecules, such as E-selectin or VCAM-1 (vascular cell adhesion molecule), and secretion of procoagulant molecules and chemokines, and these are found in different chronic inflammatory diseases, such as atherosclerosis and hypertension [74]. In general, it is accepted that endothelial cell dysfunction is a hallmark of a wide range of cardiovascular diseases [2].

Regarding the effects of SPMs in ECs, in vitro studies show that certain SPMs, such as LXA_4_, MaR1, RvE1, RvD1 and RvD2, attenuate the expression of adhesion molecules, decrease the release of proinflammatory cytokines, such as IL-12 (involved in leukocyte recruitment), and regulate leukocyte–endothelial interactions [13,75,76,77]. In addition, RvD2 enhances endothelial cell migration, but not proliferation, in a Rac-dependent manner [78] and stimulates the production of the vasodilators nitric oxide (NO) and prostacyclin (PGI_2_) [79] (Figure 5). Similarly, AT LXA_4_ directly increases plasma NO levels and decreases the number of leukocyte–endothelial interactions. Of note, evidence suggests that this NO production underlies some of the beneficial effects of aspirin in the management of cardiovascular diseases [80]. Lipoxins and AT LXs increase PGI_2_ production and block ROS generation in human ECs by downregulating NADPH oxidase [81,82]. In addition, lipoxins and their derivatives upregulate the expression of the stress-response protein heme oxygenase-1 in human ECs, suggesting a new mechanism for the anti-inflammatory effect of these mediators [83]. Lipoxins and AT LXs have further effects in ECs, such as downregulation of the endothelial activation markers VCAM-1, ICAM-1 and E-selectin [84,85]. Moreover, LXA4, 15-epi-LXA4 and other analogs limit endothelial migration and proliferation by inhibiting vascular endothelial growth factor (VEGF) and VEGF receptor expression, supporting a novel role for these mediators as angiogenesis modulators [86,87] (Figure 5). Other mechanisms responsible for the beneficial effects of SPMs on ECs are attenuation of NF-κB activation, upregulation of cAMP, which is involved in anti-inflammatory and anti-oxidant pathways [76], and activation of the GSK-3β–C/EBPβ axis, which ultimately leads to upregulation of Del-1, an endothelial cell-secreted anti-inflammatory protein [88].

#### 3.4.2. SPM Effects in VSMCs

In response to vascular injury, VSMCs switch from a contractile to a synthetic phenotype and become more proliferative and chemotactic. In this sense, acute vessel injury is characterized by inflammation, cytokine production and VSMC proliferation and migration, which ultimately lead to neointima formation and reduction in lumen diameter. Treatment with certain SPMs, such as MaR1, RvD1, RvD2, 15-epi-LXA_4_ or RvE1, attenuated VSMC proliferation both in vitro and in vivo [89,90,91,92,93] and decreased migration, cytoskeletal changes, expression of adhesion molecules (e.g., VCAM-1, ICAM-1), ROS production and release of proinflammatory mediators in several animal models [76,90,92,94] (Figure 5). In addition, the expression of ALX–FPR2 seems to be related to collagen accumulation in VSMCs, as VSMCs derived from Ldlr^−/−^ × FPR2^−/−^ double-knockout mice exhibited decreased collagen production and increased collagenase pathways [95]. Therefore, in general, SPMs have the potential to regulate vascular remodeling in different conditions (Figure 5). These anti-proliferative and anti-inflammatory effects can be explained by downregulation of NF-κB or activation of cAMP–PKA pathways [13].

The above-mentioned effects of SPMs in vascular cells clearly affect vascular structure. However, vascular function can be also modulated by these molecules, and this has been particularly explored in the pulmonary vasculature. Thus, RvE1, RvD1 and RvD2 inhibited vasoconstrictor responses induced by U46619, a TP agonist, in human pulmonary arteries (HPA) and also in rat aorta [96]. Moreover, RvE1 and RvD1 attenuated pulmonary hypertension-induced hypercontractility and Ca^2+^ hypersensitivity in cultured HPA SMCs [61,97]. A recent study showed that RvE1 also suppresses vasoconstriction of rat pulmonary arteries and HPAs, probably via inhibition of Src family kinases [98]. On the other hand, the involvement of LXA_4_ in vascular function is controversial. Both vasodilator effects through NO production [99] and vasoconstriction and endothelial dysfunction via RhoA/Rhokinase and oxidative stress production have been described [100]. In this sense, it has been suggested that LXA_4_ could participate in the development of post-angioplasty complications, such as vasospasm, in patients treated with aspirin [101].

## 4. SPMs in Cardiovascular Diseases

Vascular remodeling is a typical feature of several cardiovascular diseases. Most of the studies analyzing the effect of SPM pathways in vascular remodeling have been performed in the context of atherosclerosis, aneurysms and neointima hyperplasia (Table 1).

### 4.1. SPMs in Atherosclerosis

Atherosclerosis is widely viewed as a chronic immunoinflammatory disease, affecting vascular and peripheral blood mononuclear cells, the major players contributing to the development of this disease [102,103]. However, emerging evidence suggests that advanced atherosclerosis could also be a result of deficient resolution of inflammation [104,105], although the mechanisms that prevent resolution of inflammation in atherosclerosis remain unclear. An imbalance between proinflammatory and anti-inflammatory mediators during atheroprogression has been found [106,107]. For example, Fredman et al. [107] showed that the RvD1/LTB_4_ ratio is significantly decreased in human vulnerable atherosclerotic plaques. SPMs are also reduced in plaques of high-fat diet-fed Ldlr^−/−^ mice and the plasma of atherosclerotic patients [62,71]. Therefore, different studies have evaluated the potential anti-atherosclerotic effects of SPMs as novel approaches to current lipid lowering strategies.

In general, SPM administration promotes plaque stability by inducing a switch of macrophage phenotypes to M2-like phenotypes and by decreasing local inflammatory cytokines, ROS production and the activation of matrix metalloproteinases that break atherosclerotic plaque [108] (Table 1). For instance, exogenous administration of RvD1 to Ldlr^−/−^ mice suppressed ROS and necrosis in murine lesions, enhanced efferocytosis and fibrous caps and decreased collagenase and metalloproteinases [107]. RvD2 and MaR1 delivery prevented atheroprogression in a model of apolipoprotein E-deficient (ApoE^−/−^) mice by polarizing macrophages to a reparative phenotype and stimulating collagen synthesis in VMSCs [106]. Similar results were described with RvE1, which displays protective effects in several atherosclerosis settings, even when administered on top of statins, by mechanisms that seem to be independent of cholesterol lowering and include the reduction of inflammatory cell infiltration and pro-inflammatory cytokine secretion [109,110], inhibition of vascular calcification of smooth muscle cells and the reduction of macrophage-derived foam cell formation [111,112]. In addition, AT LXA_4_ administration blocked atheroprogression in ApoE^−/−^ mice via FPR2 receptors [91].

Genetic models of SPM pathways have provided important information on the protective effects of endogenous molecules in atherosclerosis progression. Thus, targeted deletion of the RvE1 receptor, ChemR23, is associated with atheroprogression and mediates RvE1 protective effects by modifying oxidized LDL uptake and phagocytosis in two independent hyperlipidemic murine models [113]. Interestingly, even though ALX–FPR2 expression has been identified in macrophages, endothelial cells and VMSCs in human atherosclerotic lesions [54], no consensus has yet been reached on the contribution of this receptor in different hyperlipidemic murine models. On the one hand, deletion of the murine homologue of human ALX–FPR2 decreased atherosclerosis development in Ldlr^−/−^ × Fpr2^−/−^ double-knockout mice by reducing macrophage infiltration [95]. On the other hand, Fpr2 deletion seems to promote a less stable plaque phenotype with decreased collagen content in VSMCs [95], suggesting that ALX–FPR2 signaling has a dual role in atherosclerosis development and plaque stability. In addition, other authors have shown that atherosclerotic lesions are enhanced in Fpr2 × ApoE double-knockout mice [114]. Moreover, administration of AT LXA_4_ was atheroprotective in ApoE^−/−^ mice via the FPR2 receptor [91]. More recently, it has been found that human GPR32 expression in a Fpr2×ApoE double-knockout background is associated with decreased atherosclerotic lesions and necrotic core via reduced aortic inflammation [115].

### 4.2. SPMs in Aneurysm

Aortic aneurysms are defined as focused dilations 1.5-times greater than normal aortic size [116] and are characterized by adventitial and medial inflammatory cell infiltration, ECM degradation, VMSC apoptosis and oxidative stress that leads to vascular remodeling characterized by adventitial thickening and media degradation [8,117,118,119,120]. 

Although abdominal aortic aneurysm (AAA) has been classically associated with atherosclerosis [121], increasing evidence indicates that the developing mechanisms for both diseases are different [8,120]. It has been proposed that aneurysm formation starts with the infiltration of inflammatory cells, including macrophages, T- and B-lymphocytes and neutrophils, among others, into the adventitia layer which subsequently migrate towards media layer. In the media, activated macrophages secrete matrix metalloproteinases (MMPs) and cytokines that ultimately lead to ECM degradation and VSMC apoptosis, thereby causing aortic layer degradation, which leads to progressive aortic dilatation and, ultimately, rupture [8,120]. 

As in atherosclerosis, some evidence suggests that in AAA there might be an imbalance between proinflammatory mediators and SPMs. A study carried out in patients undergoing AAA surgery, where lipid mediators were evaluated in plasma obtained from the surgical area, revealed two different profiles. One group of patients presented a pro-inflammatory profile characterized by increased levels of leukotrienes and prostaglandins and their precursors, while another group displayed a pro-resolving profile with increased levels of RvE1 and RvE2 and the RvD1 and MaR1 precursors 17-HDHA and 14-HDHA, respectively. However, they did not investigate the correlation between lipid mediator profiles and AAA progression or patient recovery [122]. Another study evaluated human aortic aneurysm samples from patients undergoing elective open surgery and aortic samples from non-aneurysmatic organ donors. The authors found lower gene expression levels of ALX–FPR2, which is the receptor for LXA_4_ and RvD1, in the adventitial layer of the aneurysmal patients compared with healthy donors [123]. This prompted investigators to determine the consequences of deleting the FPR2 gene in an experimental model of AAA. They found that ApoE^−/−^ mice lacking the Fpr2 receptor (ApoE^−/−^Fpr2^−/−^ mice), exhibited an exaggerated aortic dilation after Ang II infusion compared with ApoE^−/−^Fpr2^+/+^ mice, with elastin disruption and MMP9 expression and a decrease in collagen deposition, accompanied by an increased infiltration of neutrophils and macrophages [123]. Similar results were observed in mice lacking the enzyme involved in LXA_4_ and RvD1 synthesis, 12/15-LOX [123], indicating a possible protective role of endogenous lipid mediators in AAA development. 

Administration of different SPMs has been beneficial in several models of AAA. This is relevant, since currently there is no efficient pharmacological treatment for AAA disease. RvD1 and RvD2 inhibit AAA development in murine experimental models [124,125]. Specifically, in the model of AAA induced by Ang II administration in ApoE^−/−^ mice, RvD2 treatment decreased AAA formation by reducing local pro-inflammatory molecules, including MCP-1, IL-6, IL-1β and RANTES, and MMP2 and MMP9 activity and increased anti-inflammatory cytokines, such as IL-10. This reduction in AAA formation was attributed to macrophage M2 polarization, as no changes in T-cell or neutrophil infiltration were observed [124]. Analogous results were described with RvD1 and RvD2 treatments in the AAA experimental model of local elastase perfusion [124]. More importantly, RvD2 administration after small AAAs had formed caused a reduction in later aortic enlargement [124], thus demonstrating its potential to slow AAA progression. More recently, the same group reported that in the same models of AAA, RvD1 administration decreased AAA formation by decreasing neutrophils and neutrophil extracellular traps release (NETosis) [125]. Of note, other SPMs also showed beneficial effects with respect to AAA formation. For example, in the experimental model of AAA using elastase infusion, MaR1 upregulated the macrophage-dependent efferocytosis of apoptotic VSMCs via LGR6 receptors [126]. Together, these results likely suggest common underlying mechanisms of SPMs to improve AAA homeostasis and integrity (Table 1). 

### 4.3. SPMs in Hyperplasia

Vascular hyperplasia and restenosis are important side effects associated with endovascular interventional procedures, such as balloon angioplasty or intravascular stenting intended for revascularization, in which the endothelium is mechanically damaged. Neointimal hyperplasia is characterized by an uncontrolled proliferation of VSMCs and fibroblasts and an increase in ECM deposition in the intima layer that leads to hypertrophic inward remodeling of arteries and veins, with a thickened intima and narrowed lumen [127]. As in atherosclerosis and AAA, neointimal hyperplasia starts with infiltration of circulating inflammatory cells, including monocytes and T-lymphocytes [128], that release cytokines and chemokines that activate the differentiation of VSMCs into a proliferative phenotype and induce their migration towards the lumen, leading to vascular stenosis [129].

Evidence of the beneficial effects of SPM administration in this type of vascular remodeling have been provided in different experimental models [127] (Table 1). In balloon-injured rabbit arteries, RvD series decreased vascular remodeling by diminishing VSMC proliferation and leukocyte recruitment, probably due to a local increase in RvD1 receptor (FPR2 and GPR32) expression and endogenous biosynthesis of other SPMs [90], demonstrating the existence of positive feedforward mechanisms. Similarly, RvD2 and MaR1 decreased vascular hyperplasia and reduced VSMC proliferation and neutrophil and macrophage recruitment in the arterial wall in a mouse model of carotid artery ligation [89]. In the same experimental model, administration of AT-LXA_4_ also reduced the intima area and the intima/media ratio in wildtype mice but not in FPR2/ALX-deficient mice [130], demonstrating that these effects are due to SPM receptor signaling. Moreover, mice lacking ChemR23 presented increased intimal hyperplasia 28 days after ligation of the left common carotid artery compared with ChemR23^+/+^ mice [131]. Conversely, RvE1 administration inhibited vascular remodeling after wire-induced injury in mice femoral arteries by hindering VSMCs migration, neutrophil infiltration and T-cell trafficking and promoting macrophage polarization toward an M2-like phenotype [94]. Furthermore, not only systemic delivery of RvD1 prevented vascular damage but locally administered biodegradable gels also ameliorated vascular hyperplasia in a rat model of carotid angioplasty and in a rabbit carotid bypass model [92,93], suggesting that local, lesion-inherent effects are likely taking place in VSMC and immune-infiltrated cells. Moreover, intravenous injection of both RvD1 and PD1 improved vascular remodeling in the rat carotid artery balloon injury model through NF-κB pathway inhibition [132]. Interestingly, in a rat model of carotid angioplasty, oral administration of RvD1 ameliorated inflammation but not intimal hyperplasia, highlighting the importance of administration routes [133].

**Table 1:** Summary of in vivo experimental approaches to test the effects of SPMs in vascular remodeling. The effects of exogenous SPM administration or genetic modification of SPM receptors in macrophages (MØs) and/or vascular smooth muscle cells (VSMCs) are shown. In general, SPM administration promotes beneficial effects in vascular remodeling, whereas deletion of SPM receptors aggravates vascular disease. AT: aspirin-triggered, oxLDL: oxidized low-density lipoprotein. hGPR32 × Fpr2^−/−^ indicates overexpression of the human GPR32 in a Fpr2^−/−^ mice. M2 indicates “resolutive” macrophage phenotype.

Disease	Approach		Target Cell and Action		Main Effects	Ref.
	SPM Admin.	Genetic Modification				
**Atherosclerosis**	RvD1	-	MØ	↑ efferocytosis	Promoted plaque stability, suppressed plaque progression	[107]
			VSMCs	unaltered No.		
	RvD2, MaR1	-	MØ	polarization to M2	Reduced atheroprogression and promoted plaque stability	[106]
			VSMCs	↑ collagen synthesis, ↑ No.		
	RvE1	-	MØ	↓ foam cell infiltration	Reduced atheroprogression	[109] [110]
	-	ChemR23^−/−^	MØ	↓ phagocytosis, ↑ oxLDL uptake	Increased atherosclerotic plaque size and necrotic core formation	[113]
			VSMCs	↓ collagen ↓ proliferation		
		Frp2^−/−^	MØ	↓ infiltration	Reduced atheroprogression Impaired plaque stability	[95]
			VSMCs	↓ collagen synthesis		
	-	Frp2^−/−^	Leukocytes	↑recruitment	Enhanced atheroprogression	[114]
	AT-LXA_4_	-	MØ	↓ infiltration	Reduced atheroprogression ↓ systemic inflammation	[91]
	-	hGPR32 × Fpr2^−/−^	MØ, neutrophils, monocytes	↓ aortic infiltration	Reduced atheroprogression, necrotic core, aortic inflammation	[115]
**Aneurysms**	-	Frp2^−/−^	MØ, neutrophils	↑ aortic infiltration	↑ aortic dilation, elastin disruption and ↓ collagen deposition	[123]
	RvD1, RvD2	-	MØ	polarization to M2, ↓ pro-inflammatory cytokines	Decreased AAA formation	[124]
			Neutrophils	↓ infiltration, ↓ NETosis		[125]
	MaR1	-	MØ	↑ efferocytosis	Decreased AAA formation	[126]
**Hyperplasia**	RvD1, RvD2, RvE1, MaR1, PD1	-	MØ, neutrophils	↓ infiltration, polarization to M2	Decreased neointima formation	[89] [90] [92,93,94] [132]
			VSMCs	↓ proliferation, ↓ migration		
	AT-LXA_4_	-	VSMCs	↓ migration	Decreased neointima formation	[130]
	-	ChemR23^−/−^	Peritoneal MØ	↑ inflammatory cytokines	Increased intimal hyperplasia	[131]
			VSMCs	↓ proliferation		

## 5. Conclusions

**Figure 6:**
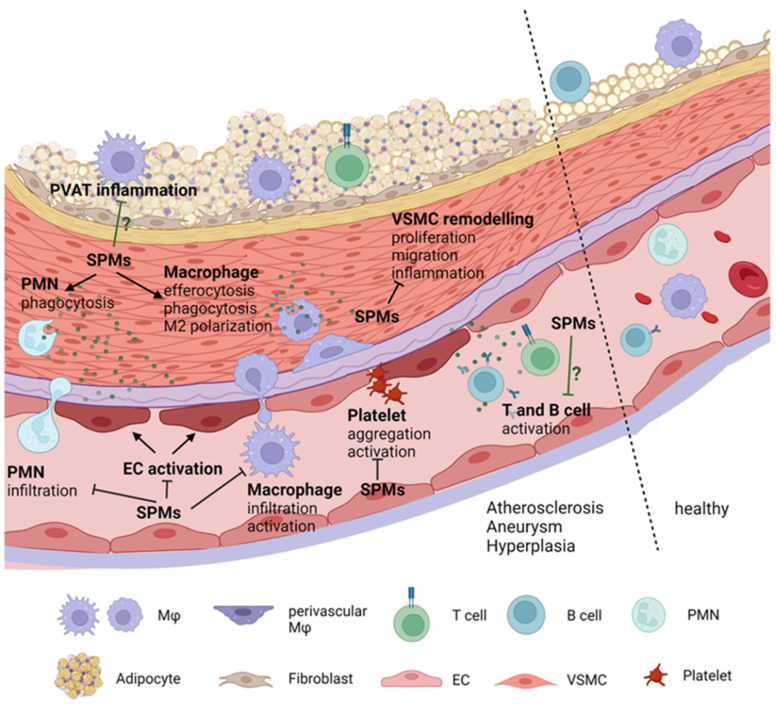
Effects of SPMs in pathological vascular remodeling. Vascular remodeling in pathological conditions, such as atherosclerosis, aneurysm and hyperplasia includes activation of endothelial cells (ECs), phenotype switching of vascular smooth muscle cells (VSMCs), immune cell (polymorphonuclear cell (PMNs), macrophage and lymphocyte) infiltration and activation, and platelet aggregation. SPMs reduce EC activation and proliferation, and migration and inflammation in VSMCs. SPMs also reduce immune cell activation and infiltration, promote macrophages M2 phenotype polarization, facilitate macrophage and PMN efferocytosis and inhibit platelet activation and aggregation.

Inflammation contributes to vascular damage in different cardiovascular diseases by activating ECs, inducing phenotypic switching of VSMCs and stimulating aberrant ECM production. This chronic inflammatory state may be caused by both an excess of proinflammatory mediators and/or by a defect or disbalance in the mechanisms of resolution, and this seems to occur in several vascular pathologies, including atherosclerosis, aneurysms and restenosis. Deletion of enzymes or receptors involved in SPM synthesis or signaling in mice has provided evidence of the beneficial effects of endogenous SPMs in vessel homeostasis, particularly in pathological situations. Moreover, exogenous administration of different SPMs improved vascular remodeling associated with disease. Notably, in general, beneficial effects are observed for different members of the SPM families, such as D or E resolvins, maresins and lipoxins, pointing to common underlying mechanisms that explain these positive effects of SPMs at the vascular level. In this sense, ECs and VSMCs express several SPM receptors that modulate fundamental processes of vascular biology, such as VSMC phenotype, the production of vasoprotective mediators and the regulation of inflammatory responses, although more research is needed to identify the molecular mechanisms involved in direct SPM actions in vascular cells. Moreover, there is no doubt that the beneficial effects of these molecules in vascular remodeling can be explained, at least in part, by effects in infiltrating immune cells. In any case, these results suggest that SPMs may offer a novel therapeutic strategy for the treatment of cardiovascular diseases associated with vascular remodeling by different mechanisms (Figure 6).

The relationship between SPMs and vascular remodeling at the clinical level has not been explored in depth, although some evidence points to impaired resolution of inflammation in some vascular pathologies. Some clinical trials using SPM precursors, such as EPA (JELIS and REDUCE-IT trials), showed cardiovascular risk reduction in patients with high triglyceride levels and high cardiovascular risk [134,135]. However, other studies found no improvement on cardiovascular events after treatment with a carboxylic acid formulation of EPA and DHA [136]. Future studies will confirm or dismiss the beneficial effects of SPMs in cardiovascular diseases and in vascular damage, either as pharmacological treatments or as predictive biomarkers of vascular disease.

## Data Availability

Not applicable.
